# Diagnostic confusion between seabather’s eruption as well as dermatophytosis and parasitic infestations

**DOI:** 10.1590/0037-8682-0462-2019

**Published:** 2020-03-16

**Authors:** André Luiz Rossetto, Catarina Cé Bella Cruz, Isabella Cruz Cesário Pereira, Juliana Arnauts Nunes, Mariana Medeiros Martins, Théo Nicolacópulos, Ana Letícia Rossetto, Vidal Haddad

**Affiliations:** 1 Universidade do Vale do Itajaí, Itajaí, SC, Brasil.; 2 Pontifícia Universidade Católica do Paraná, Curitiba, PR, Brasil.; 3 Universidade Estadual Paulista de Botucatu, Botucatu, SP, Brasil.

**Dear Editor:**

Seabather’s eruption (SBE) is an acute dermatitis characterized by itchy and erythematous papules that appear during or after bathing in the sea. It is caused by contact with the larvae and adult of the jellyfish (*Linuche unguiculata* and *L. aquila*, recently found in the Philippines) and larvae of the sea anemone *Edwardsiella lineata* ^1^ ^-^ ^3^. Limited information is available on this type of dermatitis. However, it is considered a public health concern in Brazilian coastal areas^2^ ^-^ ^7^. Here, we report two typical cases of SBE characterized by exuberant and eczematous lesions, aggravated by diagnostic confusion with dermatophytosis and parasitic infestations such as cutaneous larva migrans and scabies.

Envenomations occurred in a female and male children aged 2 and 11 years, respectively, who frequently went to the coastal beaches in the state of Santa Catarina, Brazil. The children experienced a pricking sensation in the pubic, genital, and gluteal regions during sea bathing, which progressed to pruritus and intensified at night.

The parents of the one child consulted a pharmacist, who diagnosed the condition as ringworm and recommended the use of topical ketoconazole. After 1 week of treatment, the manifestations had worsened. Thus, the parents sought consultation from a dermatologist. The other child was taken by his mother to a pediatric emergency department. The condition was diagnosed as cutaneous larva migrans, and topical thiabendazole was prescribed and used for 2 weeks. However, due to the aggravation of pruritus and lesions, the child returned to the hospital for consultation from the same pediatrician who changed the diagnosis to scabies. Topical permethrin was prescribed, but the mother subsequently decided to consult a dermatologist.

Both dermatological examinations revealed intense pruritic and eczematous, erythematous papules located mainly in the gluteal regions (Figures 1A, B, E, and F). Dermoscopic examinations did not reveal the structures of *L. unguiculata* or cnidocytes, and examination of areas with exulcerated and meliceric crusting revealed nonspecific findings (Figures 1C, D, G, and H).

**FIGURE 1:**
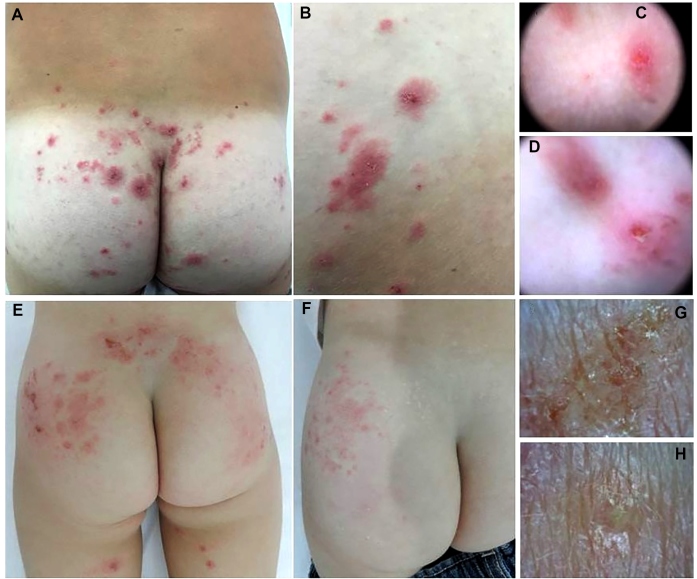
(A-D): Case 1. A: Erythematous and eczematous papules in the gluteal regions. (B): Eczematous papules and crusts. (C-D): Dermatoscopy (Dermlite DL4^®^, ×10) images of areas with diffuse and exulcerated nonspecific erythema. (E-H): Case 2. E: Erythematous and eczematous papules with erosions in the lumbar area, gluteal region, and posterior thighs. (F): Persistent lesions in the left side of the gluteal area after 15 days of treatment with hydrocortisone cream. (G-H): Dermoscopy (Dino-Lite^®^, ×10) images of areas with exulcerations, meliceric crust, and uncharacteristic edema.

The patients were treated with systemic antihistamines and corticosteroid creams. The condition of both the patients improved, with complete regression of the lesions and pruritus after 15 and 21 days of treatment, respectively.

The occurrence of SBE among individuals living on the Brazilian coast is caused by the planulae and the scyphomedusa *L. unguiculata*, which is found on the Santa Catarina coast^2^ ^-^ ^8^. Limited information is available of this type of dermatitis, and its occurrence is mainly observed in individuals living on the coastlines of São Paulo and Santa Catarina^3^ ^-^ ^7^. In this study, the initial clinical manifestations, which include erythematous, itchy papules that appeared during exposure to seawater, particularly in areas covered by bathing suits, with a gradual increase in itching, were typical of SBE in children^1^ ^-^ ^7^. 

Clinical and epidemiological diagnoses can be made. However, the absence of confirmatory laboratory examination findings can lead to diagnostic confusion^1^ ^-^ ^7^. Differential diagnoses, such as swimmer’s itch and allergic reactions to insect bites, can be distinguished based on well-characterized clinical manifestations that appear during bathing in freshwater infested by *Schistosoma* larvae and insect bites generally in areas not covered by clothing, such as the upper and lower limbs^3^ ^-^ ^7^.

Contact with sand on the beaches supported the diagnostic suspicion of ringworm and parasitic infestations, such as scabies and cutaneous larva migrans. Dermatophytosis is not characterized by intense itching and multiple erythematous papules that appear immediately after bathing in sea water. If there is confusion with the diagnosis of tinea, anamnesis and mycological examinations can be performed.

Dermoscopy is part of the routine dermatological examination and is a noninvasive examination that can help obtain an immediate and reliable diagnosis in cases of various inflammatory diseases, such as psoriasis, cutaneous sarcoidosis and lupus erythematosus, pigmented lesions, and parasitic infestations^6^. The number of dermatoscopic studies on SBE is limited, and they have revealed nonspecific findings^6^. Moreover, dermoscopy has been useful in the diagnosis of scabies because it can reveal the characteristic tunnels with small triangular areas representing the anterior part of the *Sarcoptes scabiei*. Similarly, cutaneous larva migrans had linear erythematous areas with yellowish or erythematous rounded blocks similar to the body of *Ancylostoma* spp^6^.

The diagnosis of SBE may have been underestimated because the condition spontaneously resolves within a few days or weeks in some patients. Dermatitis regresses well with treatment with systemic antihistamines and topical corticosteroids with low potency. Because the incidence of this condition is higher in children than in adults due to longer exposure to seawater, topical hydrocortisone has been the first treatment choice.

Even with nonspecific findings, dermatoscopy is useful in differentiating SBE from parasitic infestations. Inadequate diagnosis and management may complicate the evolution of the condition to exuberant and eczematous lesions. Moreover, knowledge about SBE among health professionals working in the Brazilian coastal can help prevent diagnostic confusion and late treatment.

## References

[1] Guevara BEK, Dayrit JF, Haddad V (2017). Seabather's eruption caused by the thimble jellyfish (Linuche aquila) in the Philippines. Clin Exp Dermatol.

[2] Eyer-Silva WA, Pitombo FB, Silva GAR (2018). Seabather’s eruption in Ipanema Beach, Rio de Janeiro, Brazil. Rev Soc Bras Med Trop.

[3] Haddad V, Cardoso JLC, Silveira FL (2001). Seabather’s Eruption: Report of five cases in southeast region of Brazil. Rev Inst Med Trop S Paulo.

[4] Rossetto AL, Mora JDM, Correa PR, Resgalla C, Proença LADO, Silveira FLD, Haddad V (2007). Seabather’s eruption: report of the six cases in southern. Rev Soc Bras Med Trop.

[5] Rossetto AL, Dellatorre G, Silveira FL, Haddad V (2009). Seabather’s eruption: A clinical and epidemiological study of 38 cases in Santa Catarina state, Brazil. Rev Inst Med Trop S Paulo.

[6] Rossetto AL, Chong FH, Correa PR, Morandini AC, Souza V, Haddad V (2015). Prurido do traje de banho: caso típico com exame dermatoscópico.

[7] Rossetto AL, Proença LADO (2012). Seabather's eruption: report of case in northeast region of Brazil. An Bras Dermatol.

[8] Morandini AC, Soares MDO, Matthewa-Cascon H, Marques AC (2006). A survey of the Scyphozoa and Cubozoa (Cnidaria, Medusozoa) from the Ceará coast (NE Brazil). Biota Neotropica.

